# Improving Management of Respiratory Tract Infections in Community Pharmacies and Promoting Antimicrobial Stewardship: A Cluster Randomised Control Trial with a Self-Report Behavioural Questionnaire and Process Evaluation

**DOI:** 10.3390/pharmacy8010044

**Published:** 2020-03-19

**Authors:** Diane Ashiru-Oredope, Anne Doble, Tracey Thornley, Ayoub Saei, Natalie Gold, Anna Sallis, Cliodna A M McNulty, Donna Lecky, Eno Umoh, Chaamala Klinger

**Affiliations:** 1 HCAI and AMR division, Public Health England, London SE1 8UG, UK; Anne.Doble@phe.gov.uk (A.D.); Ayoub.Saei@phe.gov.uk (A.S.); Cliodna.McNulty@phe.gov.uk (C.A.M.M.); Donna.Lecky@phe.gov.uk (D.L.); Eno.Umoh@phe.gov.uk (E.U.); 2 Pharmacy Outcomes and Research, Boots UK, Nottingham NG90 1BS, UK; tracey.thornley@boots.co.uk; 3 School of Pharmacy, University of Nottingham, Nottingham NG7 2RD, UK; 4 Behavioural Insights team, Public Health England, London SE1 8UG, UK; Natalie.Gold@phe.gov.uk (N.G.); Anna.Sallis@phe.gov.uk (A.S.); 5 Faculty of Philosophy, University of Oxford, Radcliffe Humanities, Woodstock Road, Oxford OX2 6GG, UK; 6 PHE South West, Public Health England, London SE1 8UG, UK; Chaamala.Klinger@phe.gov.uk

**Keywords:** antibiotic pharmacist, self-care, primary care, general practice, behaviour change, evaluation

## Abstract

In England, 81% of all antibiotic prescriptions originate in primary care/community settings, of which up to 20% are thought to be inappropriate. Community pharmacies are often the first point of community contact for patients with suspected infections; providing an opportunity for community pharmacy teams to promote antimicrobial stewardship (AMS). The objective of the study was to improve the management of infections and antimicrobial stewardship in community pharmacies. The study methodology included a non-blinded cluster randomised control trial with pharmacy staff in 272 community pharmacies in England. The intervention arm received an AMS webinar and a patient facing respiratory tract infection (RTI) leaflet (TARGET TYI-RTI) for use in everyday practice for four weeks. The control arm received a webinar on how to participate in the study. The primary outcome was self-reported referrals to general practitioners (GPs). The secondary outcomes were; provision of self-care advice/ written information to patients, referrals to pharmacists, sign-posting to non-prescription medicines and common barriers and facilitators to advice-giving in community pharmacies. Ethics approval was granted by the Public Health England Research Ethics and Governance Group. 66.91% (182 of 272) of pharmacies provided 3649 patient consultation data reports across both arms. Use of the leaflet was associated with a lower likelihood of referrals to GPs for certain RTIs (p < 0.05) and a more frequent provision of self-care advice than the control (p = 0.06). Opportunities to deliver self-care advice were limited due to lack of time. Pharmacy staff had good motivation and capability for managing self-limiting infections but the opportunity to do so was a perceived barrier. Use of the TARGET leaflet facilitated pharmacy staff to give more self-care advice and decreased referrals to GPs.

## 1. Introduction

Tackling antimicrobial resistance (AMR) is a UK strategic priority, with the aim of reducing the number of serious infections that are resistant to treatment [1]. Inappropriate use and overuse of antimicrobials such as antibiotics is a significant driver of antimicrobial resistance [2]. Within England, primary care settings account for the majority of antibiotic prescribing (81%). The public report that they contact their general practice because of worries about illness severity, length of illness, and concerns about complications [3]. Although antibiotic prescription numbers in this setting have declined by 13.2% over the past five years, there is still room for improvement, particularly as the number of bloodstream infections caused by resistant organisms continues to rise [4].

Recent qualitative research using the behaviour change wheel highlighted that community pharmacists see their role in antimicrobial stewardship (AMS) as providing self-care advice to patients who present with common infections and improving patients’ adherence to prescribed antibiotics [5,6]. Additionally, that resources are required to support pharmacists and pharmacy staff to provide self-care and antibiotic-compliance advice to patients [5].

The NHS encourages community pharmacy teams to support self-care, as they are often the first point of contact for patients who seek advice and support, particularly for minor ailments [7,8]. The public trust their pharmacist’s advice yet only 5.7% contacted their community pharmacist with their most recent respiratory tract infection (RTI) [3].

The TARGET “treating your infection—respiratory tract infection” (TYI-RTI) leaflet is one evaluated resource already in use within general practice, where it forms part of a wider AMS primary care toolkit. This acts as both a resource for clinicians (doctors, advanced nurse practitioners and general practice pharmacists) to use during consultations and also take-home information for patients on the usual duration of common self-limiting respiratory infections, including how to self-care, and when to seek further advice [9]. The TARGET TYI-RTI leaflet was adapted and launched for community pharmacy teams in 2015, to share with patients who are seeking advice about common self-limiting respiratory infections (Supplementary Information 1) [10]. This is the first time its use in community pharmacy is being assessed.

We aimed to test an intervention to improve the management of respiratory tract infections and promote antimicrobial stewardship within a community pharmacy. The objectives included evaluating the effect of providing community pharmacy staff with webinar training in AMS, combined with printed copies of the TARGET TYI-RTI leaflet on the resulting number of GP referrals and the provision of self-care advice. We also assessed the barriers and facilitators to providing self-care advice in community pharmacy and conducted a process evaluation of the project.

## 2. Materials and Methods

### 2.1. Study Design

Setting: Independent and multiple/chain community pharmacies in urban and rural areas in the South West of England and one locality, Southwark, London.

Study design: The study was a two-armed non-blinded cluster randomised control trial with the community pharmacy as the unit of randomisation.

The primary outcome was self-reported referrals to GPs (primary care doctors). Secondary outcomes were; the provision of self-care advice and written information to patients, referrals to pharmacists (by other members of pharmacy support staff), sign-posting to non-prescription medicines (i.e., medicines that can be purchased over the counter at the pharmacy without a prescription from a doctor) and uptake of resources (Supplementary Information 2 and 3). Additional data were collected to understand the experiences of participating in the study and common barriers and facilitators to advice-giving in community pharmacies.

An online survey was conducted to understand the influences on pharmacy team members’ behaviour in managing self-limiting infections, in addition to a self-reported questionnaire informed by the COM-B (capability, opportunity, motivation and behaviour) model [6,11]. Following the consultation data collection period, pharmacy teams in the control and intervention pharmacies were invited to complete identical surveys, including a 25-item behavioural assessment questionnaire to be completed by at least two participating staff and a process evaluation questionnaire to be completed by the lead contact (Supplementary Information 4 and 5). The questionnaire was a cross-sectional survey, designed using the COM-B model [8,11] with questions covering the capability (7 questions), opportunity (3 questions), motivation (6 questions) and behaviour (9 questions) of pharmacy staff in the delivery of self-care advice around common infections, with focus on antibiotic resistance and antibiotic use. The process evaluation questions covered study participation on the ease of participation and data recording, with opportunity to comment more generally on what worked well or less well in the study. Figure S1 illustrates a flow chart of activities during the study period.

The study was conducted in accordance with the Declaration of Helsinki, and the protocol was approved by the PHE Research Ethics and Governance Group (REGG). (Project identification code: R & D 362).

### 2.2. Participants: Recruitment and Intervention Allocation

All community pharmacies in the South West region of England (n = 841) and one locality, Southwark in London (n = 62), were eligible and invited to participate in the study. Local Pharmaceutical Committees (LPCs) sent an invitation letter via email to the local pharmacies, which included an information leaflet and link to an online expression of interest form. Pharmacies were offered a £50 incentive for participation to recognise the additional time involved.

Community pharmacies who expressed interest were subsequently stratified by geographical locality and independence/multiple (or chain) status (see Box 1). Pharmacies within two strata (either urban or rural localities) were independently randomly allocated to either the intervention or control arm. Individual pharmacies formed the unit of the intervention allocation and data analysis of patient consultation outcomes. This arrangement, therefore, took account of the clustering effect by individual pharmacies (block cluster randomisation). The analyst was blinded to the study allocation for analysis purposes. The pharmacy details available to the statistician were non-informative for the identification of exact pharmacies.

Box 1 **Local pharmaceutical committees and types of pharmacy.**

Local pharmaceutical committees (LPCs; organisations that represent all NHS community pharmacy contractors in a defined locality).

An independent pharmacy is a retail pharmacy that is not directly owned (or operated) by a publicly traded company. Generally, large multiples/chains are those with 100 or more pharmacies; small multiples/chain are with 6–99 pharmacies and independents own 1 to 5 pharmacies.

Intervention arm: intervention pharmacy staff received webinar-based AMS training in January 2018 and were provided with 50 printed copies of the TARGET TYI-RTI community pharmacy leaflet. Pharmacies obtained additional copies either by request or from the study group or by photocopying the leaflets.

Control arm: control pharmacy staff were invited in January 2018 to participate in a live webinar focused on informing them of the process for participating in the study (e.g., data collection requirements). Control arm pharmacies continued with normal practice throughout the study period. In this context, regular interaction with patients concerning minor conditions and management of infections were not altered. Printed TARGET TYI-RTI leaflets were not provided to the pharmacies, but data collection was still required.

Following recruitment, two reminder emails were sent to the project leads in both study arms about the available webinars for cascade to relevant pharmacy staff. Detailed briefing notes on participating in the study were also provided to all pharmacies (Supplementary Information 2). Recordings of both the intervention group [12] and control group [13] webinars were also available online for those not able to attend the live webinar. 

The URL to the behaviour questionnaire was sent to the project lead in each pharmacy for cascade to pharmacy staff that had been involved in providing consultation data. The process evaluation questionnaire was to be completed by the project lead.

Patient consultation inclusion criteria: any pharmacy-based consultations between pharmacy staff and patients (of any age) presenting with common self-limiting RTIs, including sore throats, common colds (or a ’runny nose’), sinusitis, coughs (or bronchitis) and middle ear infections.

Patient consultation exclusion criteria: patients who visited the pharmacy to request specific medicinal product(s) without seeking advice; patients attending with a prescription for antibiotics; and those attending for self-care advice for any other non-RTI related reason.

### 2.3. Data Collection

Pharmacy staff in both control and intervention arms were asked by the study team to collect data from RTI patient consultations that met the inclusion criteria, over a two-week period (22 January until 4 February 2018), using a printed copy of the data collection tool provided by the study group (Supplementary Information 3). Pharmacists were asked to input this data into the ‘Consultation Data Input Sheet’ after each consultation or during the day, and transfer this onto an online form via the PharmOutcomes^®^ portal (an online data recording tool) at the end of each working day. Information collected included patient demographics (age group estimate—child, teenager, adult, elderly; gender), type of RTI and consultation outcome. Possible recorded consultation outcomes included referral to a GP or other health service (primary study outcome); provision of self-care advice; provision of written information; sign-posting to non-prescription/over-the-counter (OTC) medicines; referral to a pharmacist (where an initial consultation involved pharmacy support staff); or referral to another health service (e.g., hospital accident and emergency department). Data on the type and location of the pharmacy, and on the staff member conducting the consultation, were also gathered.

The initial study period was extended by two weeks following requests from participating pharmacies to allow for additional time for data collection, due to it being a very busy period and reported under-staffing in many of the participating pharmacies.

### 2.4. Statistical Analysis

A descriptive analysis of demographic data from patient consultations was performed to compare the study arms. This did not account for clustering (by individual pharmacies). A logistic mixed-effects model(s) to account for clustering by pharmacies was conducted to analyse the effect of the intervention on each outcome. The model includes extra variables in addition to the intervention, to control for possibly confounding variables that might influence the association between intervention and the outcomes. The model also included the providers as random effects, to consider the extra variation that was not explained by explanatory variables, of which were the following: patient age group, sex, RTI type, pharmacy staff type and date of consultation, as well as alternative consultation outcomes (besides the outcome of interest). All data analysis was performed using Microsoft Excel^®^ and STATA^®^ (version 13). The COM-B and process evaluation questionnaire results were summarised using standard descriptive statistics.

## 3. Results

### 3.1. Summary of Data Collection

A total of 272 out of 903 community pharmacies (30%) expressed an interest in the study. Of these, 182 pharmacies submitted consultation data (67% completion rate); 92 of these had been randomised to the intervention group and 90 to the control group. A summary of consultation data collected in this study and a comparison of study arms are outlined in Table 1, Table 2 and Figure S2. Intervention and control arms were not significantly different in terms of independence status, however data collection was significantly higher in the control arm. The average number of individual patient consultation data forms submitted per pharmacy were 13.5 and 15.5 for the intervention and control arms respectively (interquartile range = 9–22 and 10–27 respectively).

### 3.2. Effect of Use of the TARGET Leaflet on Consultation Outcomes

**Figure 1:**
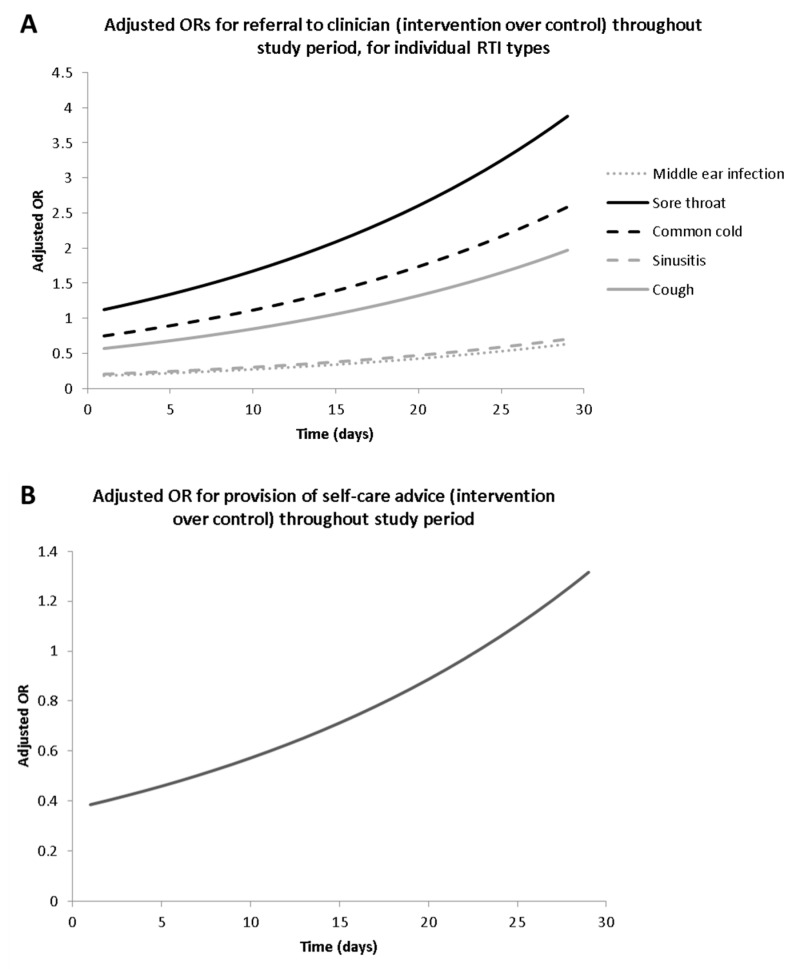
The relationship between the intervention and outcomes over time—(a) referral to doctor (by individual respiratory tract infection (RIT) type), (b) provision of self-care advice.

Statistical modelling demonstrated that the relationship between the intervention arm and referrals to a doctor or provision of self-care advice outcomes was complex, exhibiting an interaction with both time (date of patient consultation in the study period) and RTI type (Figure 1). The alternative model’s analysis provided evidence to suggest that the intervention was associated with a decrease in the likelihood of referral to a GP (adjusted odds ratio (aOR) = 0.91), when all factors were held constant; although this effect was greater during the early period of the study (defined as the first two of the four weeks) (Figure 1) and notable for middle ear infections (aOR = 0.18; p ≤ 0.001), sinusitis (aOR = 0.20; p ≤ 0.001) and possibly coughs (aOR = 0.54; p = 0.13), but not sore throat (aOR = 1.07; p = 0.89) or common cold (aOR = 0.72; p = 0.50). The intervention was also associated with the increased provision of self-care later in the study period, which was defined as the last two weeks (Figure 1). For consultations by non-pharmacists, there was limited evidence to suggest that patients in the intervention arm may have been referred to a pharmacist more frequently than those in the control arm (OR = 1.42; p = 0.11).

Patient gender did not influence the likelihood of referral to a GP. Looking within specific patient age groups, adults were more likely to be referred to a GP than children (OR = 1.58; p = 0.05). By comparison, both teenagers (OR = 0.55; p = 0.04) and adults (OR = 0.47; p < 0.01) were less likely to be referred to a pharmacist than a child.

The results of the possible multiple secondary outcomes from a single consultation (e.g., patients consulting may have received both self-care advice and written information) highlighted that patients who were referred to a pharmacist by other pharmacy staff were more likely to then be referred to a GP by the pharmacist than the patients who were not referred to a pharmacist (OR = 5.47; p ≤ 0.001). Conversely, patients who received self-care advice or written information, or were recommended non-prescription medicines, were less likely to be referred to a GP than patients who did not receive self-care advice or written information, or who were recommended non-prescription medicines. Patients were more likely to receive written information (including, but not exclusively the TARGET leaflet) if also provided with self-care advice (OR = 3.53; p < 0.01). Patients for whom non-prescription products/medicines were recommended were more likely to receive self-care advice (OR = 2.23; p < 0.00) and were less likely to receive GP referral (OR = 0.07; p < 0.01) than patients who were not recommended non-prescription products.

### 3.3. Questionnaire to Assess Capability, Opportunity, Motivation and Behaviour (COM-B) around Provision of Self-Care Advice

Questionnaire respondents included healthcare counter staff, store managers, and pharmacists. Response rates and demographics were similar for both study arms. Overall, 296 questionnaires were completed by staff in 157 participating pharmacies, across both study arms (86% response rate overall) (Figure S2).

Notably, 84.7% of respondents worked in a pharmacy multiple. The median length of pharmacy experience among individual respondents in the control arm was 8.5 years (IQR = 3–20). Only 13.9% (22% of pharmacists and 7% of non-pharmacist staff) reported having previously undertaken training or continued professional development in antimicrobial resistance or a related topic in the previous year, with no difference between study arms; 6% of all respondents had been involved in an antimicrobial stewardship project within the last year.

Results from both the intervention and control arms for the COM-B analysis have been combined, as no significant difference in responses were found between groups (Table 3 and Table 4).

Capability: Nearly all respondents (94.9%) agreed or strongly agreed that they knew what self-care advice to give; 1.7% disagreed or strongly disagreed. However, 25.3% agreed or strongly agreed that they found it difficult to explain to patients that they should not have antibiotics for common infections.

Opportunity: Many (40.8%) agreed or strongly agreed that they did not have time to give the advice they wanted, due to other pressures (28.7%, disagreed or strongly disagreed and 30.4% were undecided), but 79.1% agreed or strongly agreed that they felt supported to give self-care advice to patients.

Motivation: Three quarters of respondents (74.0%) agreed or strongly agreed that they played a key role in helping to control antibiotic use and 95.3% responded that it was important for them to give self-care advice for common infections.

Behaviour: A third of respondents (33.5%) reported that self-care advice/resources were often or very often given to patients. More than a third (38%) reported that they often or very often would have liked to give a patient/customer self-care resources, information or advice, but were unable to. The mean number of self-care conversations per person per day was 7.2, with resources being given out a mean of 6.4 times per person per day. Staff reported an average of 3.3 occasions per day when they would have liked to give a patient/customer self-care resources, information or advice, but they were not able to.

### 3.4. Process Evaluation Key Findings

Process evaluation questionnaires were completed by 156 individual pharmacies and 177 staff members (similar numbers of forms were submitted from both study arms). The majority of completed evaluations estimated that data was collected on between 25%–75% of eligible patient consultations (72.9% process evaluations; 31.6% estimated > 25%—<50% coverage and 41.2% estimated 75% coverage). Non-completion was mainly due to competing tasks (63.3% staff ‘too busy’ or 11.9% ‘other things more important’) or forgetfulness (54.8% identifying it as a reason for non-completion); only 5.7% identified ‘lack of information’ as a factor responsible for non-completion.

Process evaluation findings also demonstrated that pharmacy staff found the TARGET TYI-RTI leaflet useful as a tool to support self-care conversations and that provision of a printed leaflet supports the integration into standard pharmacy practice. The majority of positive comments about the intervention referred to the quality and content of the leaflet, its utility in facilitating self-care conversations, and the benefits of the leaflet as a resource for patients to take home. Some respondents suggested that the leaflet could be made shorter or easier to read.

## 4. Discussion

This study suggests that providing a short webinar training to pharmacy staff on AMS along with instructions on using the TARGET TYI-RTI patient leaflet, and provision of printed copies of the leaflet in community pharmacies were associated with a greater provision of self-care advice and reduced likelihood of GP referrals for infection compared to the control group. Efficacy was greatest in the two weeks after the webinar, possibly linked to staff being more motivated immediately after training. Patients in the intervention arm were more likely to receive written information if also provided with self-care advice, suggesting that the leaflet facilitated self-care for advice.

These interventions have the potential to support the government’s ambition to reduce inappropriate antibiotic consumption [1], particularly since interventions that help to both reduce referrals to GPs and inform patients on the appropriate use of antibiotics (including when they are necessary or unnecessary) may reduce patient expectation to receive antibiotics for self-limiting infections, and in turn reduce pressure on GPs to prescribe antibiotics when they are not needed [14]. The leaflet, which is available at no cost from the TARGET website (www.rcgp.org.uk/targetantibiotics), can support efforts to raise awareness on the appropriate use of antibiotics, use of available resources, and signpost to sources of advice and information (for instance, directing patients and customers to the Antibiotic Guardian website, a pledge-based campaign website) (www.antibioticguardian.com). It also supports the provision of clear and consistent messages that reinforce patient-pharmacist conversations.

This study also supports recommendations in the “Stay Well” pharmacy campaign, for pharmacies to be the first port of call for patients with minor ailments [15]. Community pharmacy teams could also play a vital role in supporting winter preparedness through widespread provision of flu immunisation; supporting the public with prevention of illness during the winter months and contributing to tackling antimicrobial resistance [7,8,16].

Use of the TARGET TYI-RTI leaflet appeared to have no significant effect on the likelihood of GP referrals amongst patients with sore throats. This may be attributed to previous interventions, including training within community pharmacies to deliver screening and treatment services for sore throats [17]; staff may already be well trained on addressing sore throats and therefore less likely to refer patients to GP. In contrast, the intervention was associated with lower GP referrals in patients presenting with several other RTI types (middle ear infections and sinusitis).

In the intervention group, there was a lower frequency of GP referrals amongst patients who also received self-care advice or written information, or were recommended non-prescription medicines, compared to the control group. This may be linked to the intervention, improving staff confidence to make an informed decision on the severity of disease and knowing where to appropriately refer. Patients who were referred to the pharmacist by pharmacy staff in the intervention arm were more likely to be referred to the GP by the pharmacist. This may be due to increased confidence from training received by or use of the leaflet by pharmacy staff in the intervention arm.

Results from behavioural questionnaires showed that pharmacy staff have good self-reported capability and motivation for giving self-care advice. Although the vast majority of staff reported knowing what self-care advice to give, about a quarter found it difficult to explain to customers that antibiotics are unnecessary for common RTIs, which is plausibly because they find it hard to explain antimicrobial resistance in lay terms. However, opportunity was a bigger barrier. More than a third of pharmacy staff reported that they lack opportunity to give advice because of other time pressures. It is possible that the TARGET TYI-RTI leaflet and training webinar can overcome barriers to self-care advice provision, by triggering conversations and facilitating a short consultation (opportunity) and enabling them to explain antimicrobial resistance succinctly in self-care conversations with patients (capability), thus being useful in time-constrained situations.

### Study Strengths and Limitations

Support from the local pharmaceutical committees during the initial design of the study and being able to gain engagement from pharmacies were both important aspects contributing to the success of the research.

The evidence base for the delivery of self-care interventions and antimicrobial stewardship in community pharmacy settings is limited and therefore needs to be strengthened [8]. To the authors’ knowledge, at the time of conducting this study, it was the first large scale AMR RCT intervention conducted in the community pharmacy setting, also evidenced from the systematic map of evidence in community pharmacy, 2000 to 2017 [6]. The use of an RCT adds to the body of evidence for AMS activities in community pharmacy.

The study had a large sample size, as one-third of pharmacies within the geographical areas were recruited to the study. However, there may have been selection bias as those agreeing to participate may have been more motivated and capable in AMS than non-participants. Although consent was given by pharmacy managers rather than the participating counter staff, the 67% data collection response rate was considered by the research team to be excellent for an evaluation in routine clinical practice. Implementation only lasted a single month. However, this was during the winter RTI season; a very busy period for community pharmacies, which may have impacted on their capacity to participate, and a third of staff reported difficulty finding time to discuss self-care with patients, conversely this does reflect usual practice of pharmacy consultations.

As control pharmacies knew they were part of a study and were asked to self-record information on their consultation, this may have increased the frequency at which advice was given. Also, the pharmacy TARGET TYI-RTI leaflet has been available electronically via the TARGET website since 2015, potentially leading to contamination if, e.g., pharmacists worked in both control and intervention groups during the study and cascaded the intervention to a pharmacy in the control group.

Finally, the patient consultations and behavioural questionnaires were self-reported rather than from an audit trail. Future evaluations could consider a McNulty–Zelen design and collect routine outcome data [18].

## 5. Conclusions

The TARGET TYI-RTI leaflet can help improve the management of infections in community pharmacies and enhance antimicrobial stewardship practice by enabling pharmacists and pharmacy staff to have infection related self-care conversations with patients. It can also contribute to increasing appropriate use of NHS resources and potentially reducing pressure on general practitioners (GPs)/doctors. The study findings indicate that the TARGET TYI-RTI community pharmacy leaflet would be a useful addition to the existing tools for national AMS campaigns, such as, “Stay Well Pharmacy”, or the “Keep Antibiotics Working” campaigns.

Pharmacy staff could use the webinar (open access) and TARGET TYI leaflets to enhance the public facing AMS advice and management of infection available through community pharmacies. Pharmacy educators may also find it useful to consider the webinar training as part of the training on management of self-limiting infections and antimicrobial stewardship.

## Figures and Tables

**Table 1:** Comparison of pharmacy types between study arms.

Pharmacy Type	Intervention (n = 92)		Control (n = 90)	
	n	%	n	%
Independent pharmacy (n = 35)	17	48.6	18	51.4
Multiple pharmacy (n = 147)	75	51.0	72	49.0

n = number of individual pharmacies; % = proportion of each pharmacy type between study arms.

**Table 2:** Comparison of pharmacy/patient characteristics and type of RTI between study arms.

	Intervention (n = 1726)		Control (n = 1923)		Chi^2^
	n	%	n	%	
Pharmacy characteristics					
Pharmacy type					
Independent	390	22.6	504	26.2	p < 0.001
Multiple	1336	77.4	1418	73.8	
Staff member conducting consultations					
Healthcare counter staff	1175	68.1	1377	71.6	p = 0.02
Pharmacist	551	31.9	546	28.4	
Patient characteristics					
Patient age group					
Child	191	11.1	234	12.2	p = 0.042
Teenager	80	4.6	104	5.4	
Adult	1,014	58.8	1167	60.7	
Elderly	441	25.6	418	21.7	
Patient gender					
Female	963	55.8	1008	52.4	p = 0.124
Male	749	43.4	898	46.7	
Unknown	14	0.8	17	0.9	
Type of RTI					
Common cold	575	33.3	683	35.5	p = 0.162
Cough	767	44.4	857	44.6	p = 0.938
Middle ear infection	95	5.5	108	5.6	p = 0.883
Sinusitis	188	10.9	239	12.4	p = 0.149
Sore throat	430	24.9	522	27.2	p = 0.125

n = number of individual patient consultations (total = 3649); % = number of consultations as percentage of total number of consultations per study arm.

**Table 3:** Capability, Opportunity, Motivation and Behaviour (COM-B) questionnaire results: 5-point Likert scale from Strongly disagree (1) to Strongly agree (5).

COM-B Dimension and Question	Score				
	1	2	3	4	5
Psychological Capability					
I know the signs and symptoms which should prompt a patient to get urgent advice from a health professional	0.7%	0.7%	2.7%	29.7%	**66.2%**
I know how long the symptoms of common infections usually last	0.3%	1.0%	7.1%	40.5%	**51.0%**
I know what self-care advice to give to patients with common infections	1.0%	0.7%	3.4%	26.7%	**68.2%**
I know what antibiotic resistance is	1.0%	1.0%	5.1%	22.0%	**71.0%**
There is a connection between giving self-care advice and reducing antibiotic resistance	0.7%	1.4%	7.8%	32.8%	**57.4%**
I find it easy to give self-care advice to patients/customers who present with common infections	0.7%	1.0%	6.1%	36.5%	**55.7%**
I find it difficult to explain to patients/customers why they should not have antibiotics for common infections	33.1%	**23.7%**	17.9%	12.5%	12.8%
Reflective motivation					
I am confident that I can give self-care advice even when I feel under pressure	1.0%	3.7%	7.8%	43.2%	**44.3%**
I believe that patients/customers will make fewer GP appointments for common infections as a result of my self-care advice	0.7%	3.7%	23.0%	**44.6%**	28.0%
Patients/customers will make GP appointments for common infections regardless of what I say	2.0%	4.7%	19.3%	36.2%	**37.8%**
It is important that I give self-care advice for common infections	2.4%	31.1%	**32.1%**	26.7%	7.8%
When patients/customers are in a hurry to leave the pharmacy, they miss out on receiving self-care advice	2.4%	9.1%	19.9%	**40.9%**	27.7%
Physical opportunity					
I have easy access to the materials I need to give self-care advice	0.3%	6.4%	16.2%	37.2%	**39.9%**
I don’t get the opportunity to give all the advice I want to give because of other time pressures	11.8%	16.9%	**30.4%**	28.0%	12.8%
Social opportunity					
I feel supported to give self-care advice to patients/customers	1.0%	3.4%	16.6%	**44.6%**	34.5%

Median response category for each question marked in bold (n = 296); modal answer to each question in bold.

**Table 4:** Behaviour questionnaire results: (a) 5-point Likert scale from Never (0) to Very often (4), median response category for each question marked in bold, (b) summary statistics for numerical free response question (n = 296).

Behaviour	a (%)					b (Distribution)			
	0	1	2	3	4	Me	M	R	IQR
Have a conversation about self-care with a patient/customer?	0.0	5.4	40.9	**38.9**	14.9	5	7.2	0–80	2–10
Give out self-care resources, information or advice to a patient/customer?	1.7	17.6	**47.3**	24.7	8.8	4	6.4	0–50	2–8
Would you have liked to give a patient/customer self-care resources, information or advice but were not able to?	8.8	29.4	**37.8**	18.9	5.1	2	3.3	0–50	0–4
Refer a patient/customer presenting with symptoms of a common infection to the GP	9.5	**45.6**	38.2	6.1	0.7	1	1.5	0–20	0–2

a: On a typical day, how often did you [do the behaviour]; b: On a typical day, please estimate how many times [you did the behaviour]; IQR = Inter quartile range; M = Mean; Me = Median; R = Range. Modal answer to each question in bold.

## Supplementary Materials

The following are available online at https://www.mdpi.com/2226-4787/8/1/44/s1, Supplementary information 1: TARGET Treating your infection RTI leaflet, Supplementary Information 2: Briefing Notes (Intervention and control group), Supplementary Information 3: Consultation Data Input Sheet, Supplementary Information 4: Behavioural assessment questionnaire, Supplementary Information 5: Process evaluation questionnaire, Figure S1: Summary of study activities, Figure S2: Data collection and responses from pharmacies in intervention and control arms, CONSORT 2010 checklist of information to include when reporting a randomised trial*.
