# Supplementary material for: Improving Management of Respiratory Tract Infections in Community Pharmacies and Promoting Antimicrobial Stewardship: A Cluster Randomised Control Trial with a Self-Report Behavioural Questionnaire and Process Evaluation
Source: Pharmacy (Basel). 2020 Mar 19;8(1):44. doi: 10.3390/pharmacy8010044 (PMC7151711; doi:10.3390/pharmacy8010044)
Supplement: Supplementary file 1 [file pharmacy-08-00044-s001.pdf]

Supplementary information 1: TARGET Treating your infection RTI leaflet

Public Health England

Self-care guide to help you treat your infection

Crystal Mark 20454 Clarity approved by Plain English Campaign

**Patient Name**		**Self-care advice provided**	☐
**Product(s) suggested/supplied**		**Patient advised to contact GP**	☐

Your infection	Usually lasts	How to treat yourself better for these infections, now and next time	When should you get help: Contact your GP practice or contact NHS 111 (England), NHS 24 (Scotland dial 111), or NHS Direct (Wales dial 0845 4647)
☐ Middle-ear infection	4 days	• Have plenty of rest.<br>• Drink enough fluids to avoid feeling thirsty.<br>• Ask your local pharmacist to recommend medicines to help your symptoms or pain (or both).<br>• Fever is a sign the body is fighting the infection and usually gets better by itself in most cases. You can use paracetamol (or ibuprofen) if you or your child are uncomfortable as a result of a fever.<br>• Other things you can do suggested by GP or nurse:<br>...<br>...	**1. to 8. are possible signs of serious illness and should be assessed urgently.**<br>**Phone for advice if you are not sure how urgent the symptoms are.**<br>1. If you develop a severe headache and are sick.<br>2. If your skin is very cold or has a strange colour, or you develop an unusual rash.<br>3. If you feel confused or have slurred speech or are very drowsy.<br>4. If you have difficulty breathing. Signs can include:<br>o breathing quickly<br>o turning blue around the lips and the skin below the mouth<br>o skin between or above the ribs getting sucked or pulled in with every breath.<br>5. If you develop chest pain.<br>6. If you have difficulty swallowing or are drooling.<br>7. If you cough up blood.<br>8. If you are feeling a lot worse.<br>**Less serious signs that can usually wait until the next available GP appointment:**<br>9. If you are not improving by the time given in the 'Usually lasts' column.<br>10. In children with middle-ear infection: if fluid is coming out of their ears or if they have new deafness.<br>11. Other ....
☐ Sore throat	7 days		
☐ Common cold	10 days		
☐ Sinusitis	18 days		
☐ Cough or bronchitis	21 days		
☐ Other infection:<br>..............................	.... days		

- Colds, most coughs, sinusitis, ear infections, sore throats, and other infections often get better without antibiotics, as your body can usually fight these infections on its own.
- The more we use antibiotics, the greater the chance that bacteria will become resistant to them so that they no longer work on our infections.
- Antibiotics can cause side effects such as rashes, thrush, stomach pains, diarrhoea, reactions to sunlight, other symptoms, or being sick if you drink alcohol with metronidazole.

Find out more about how you can make better use of antibiotics and help keep this vital treatment effective by visiting and pledging at **www.antibioticguardian.com**

Never share antibiotics and always return any unused antibiotics to a pharmacy for safe disposal

NHS Scotland · Royal Pharmaceutical Society · PharmacyVoice · BSAC The British Society for Antimicrobial Chemotherapy · RCGP Royal College of General Practitioners · UKCPA Clinical Pharmacy Association · Royal College of Nursing The voice of nursing in the UK · IPS Infection Prevention Society · BIA British Infection Association

This leaflet for community pharmacy was developed in collaboration with these professional societies.

Supplementary Information 2 – Briefing Notes (Intervention and control group)

Antimicrobial stewardship and self-care advice in pharmacies

- **NB: Please Do not move leaflets to other pharmacies in which you work**
- **If you run out of materials whom to contact- XXXXX on 0300 xxxx xxxx option 1, then option 1 again**

Briefing Note- Group A (Intervention Group)

About the project

Tackling antimicrobial resistance (AMR) is a key public health priority. Inappropriate use and overuse of antimicrobials such as antibiotics is a major driver of antimicrobial resistance. Community Pharmacy teams have a key role in contributing to tackling AMR, public health and promoting healthy living with the public. The project aims to assess the use of the TARGET community pharmacy leaflet as an integrated core part of your team's everyday practice for two weeks. This intervention will be evaluated by a randomised controlled trial. It is hoped that this project will generate evidence on the vital role played by Community Pharmacy Teams within public health. This project will also help provide evidence and contributes to Healthy Living Pharmacies, Making Every Contact Count and improving quality. Pharmacies can use the data collection as evidence of audit.

Who is doing the study?

The research leads for this study are Drs Diane Ashiru-Oredope and Chaamala Klinger. It is funded by the Public Health England Antimicrobial Resistance Programme and will take place in the South West of England and in Southwark.

What do you need to do?

The project aims to assess the use of the TARGET community pharmacy leaflet as an integrated core part of your team's everyday practice for two weeks. We'll therefore need you and your team to use this leaflet to support appropriate self-care conversations during this period and capture some simple data about these. We'll then ask you to review your experiences. We've outlined the steps we need you and your team to take below:

- Watch the short (15-minute) webinar which will teach you about antibiotic resistance, the TARGET leaflet and how to use it.
- Use the TARGET leaflet on patients/customers with any of the following common infections:
    - Middle ear infection
    - Sore throat
    - Common cold/runny nose
    - Sinusitis
    - Cough or bronchitis

- Collect data on all conversations that are symptom led about self-care advice for the following ailments
    - Middle ear infection
    - Sore throat
    - Common cold/runny nose
    - Sinusitis
    - Cough or bronchitis

      during the two weeks beginning 22nd and 29th January 2018. This should take a few mins per day as there is no data being collected that you wouldn't have already obtained as part of normal conversations with the patient/customer. A Consultation Data Input Sheet has been provided to aid data collection and data should be submitted using the PharmaOutcomes Portal.

- Complete questionnaires about your experience of giving self-care advice to people with common infections which should take no more than 10 mins.

Who has been invited to participate?

- All patient/customer facing staff in pharmacies in Avon, Cornwall, Devon, Gloucestershire, Somerset and Southwark are invited to take part.

Which patients/customers are included in the study?

Any patient/customer coming to your pharmacy who asks you for advice with any of the following infections:

- Middle -ear infection
- Sore throat
- Common cold/runny nose
- Sinusitis
- Cough or bronchitis

Which patients/customers are not included in the study?

- Patients/customers coming to the pharmacy with the respiratory tract or associated infections mentioned in the list, who have a prescription for antibiotics
- Patients/customers purchasing products to manage the symptoms of their infections without asking for advice.
- Patients/customers coming to the pharmacy for any other reason such as self-care for other conditions (for example back pain, constipation etc.)

What are the timelines for the project?

**Action**	**Commence week beginning**
You will receive the link to the webinar	2nd Jan 2018
Webinar is available	8th Jan and 15th Jan 2018
Use TARGET leaflets in your conversations with patients/customers, and complete Consultation Data Input for 2 weeks	22nd and 29th Jan 2018

You'll be sent the evaluation questionnaires	5th Feb 2018
Deadline for questionnaire completion	18th Feb 2018

What should I be aware of when completing the Consultation Data Input Sheet?

A Consultation Data Input Sheet has been provided to aid data collection and data should be submitted using the PharmOutcomes Portal. The key definitions and explanations are listed below. These definitions can also be found in the Consultation Data Input Sheet.

Consultation: Any verbal exchange with a patient/customer about symptoms of the common infections listed above. This may include minimal contact such as a simple request for a cough medicine

Age group code: C: Child; T: Teenager; A: Adult; E: Elderly

OTC Meds: Over the counter medications given/purchased.

Advised to see GP/ Out of hours/ A and E-

"No" indicates either

- you did not mention the GP or
- you advised the patient/customer not to see the GP if they asked you or
- you suggested they should see them only if things do not improve.

"Yes" indicates you suggested the patient/customer should contact the GP straight away.

Yes GP= GP

Yes OOH service= OOH

Yes A and E= AE

Self-care advice given-"Yes" indicates you suggested simple measure that patients/customers can take to care for themselves; for example taking plenty of rest. Dosage or compliance advice is not included. "No" indicates no advice on these matters was offered.

Leaflet given- Did you give a leaflet about antibiotics, infections or self-care for them to take home? If “yes”, what is the name of the leaflet you used?

When should I complete the Consultation Data Input Sheet?

It is best to complete the data input sheet each time you have a consultation. This will help to capture the data accurately and ensure that all of the information is captured. Data should be submitted using the PharmOutcomes Portal. Remember to ensure that no patient/customer-sensitive information, such as names or addresses are included. All data should be added to PharmOutcomes by 9th February 2018.

When should I complete the Post-Study Questionnaire?

Please complete the questionnaire as soon as possible when you receive it and before the 18th of February. **If you have been away on annual leave during the study period, please do not complete the questionnaire.**

Will the information obtained in the study be confidential?

In accordance with the Data Protection Act 1998, data will be kept confidential as follows: Data will be collected via questionnaires and data entry forms. Data will be stored on PharmOutcomes and PHE secure servers. No identifiable data will be collected.

What will happen to the results of the study?

- Summary of findings will be included in the 2018 national English Surveillance Programme for Antimicrobial Utilisation and Resistance (ESPAUR) report. Full findings will be submitted for peer review publication. We will provide a 1 page summary of findings to all participating pharmacies.

Do I have to take part?

- Taking part is entirely voluntary, and all participants will be asked to sign a consent form.

What are the advantages/benefits and disadvantages/risks of taking part?

The data being collected should be part of a normal consultation with the patient/customer who comes in with a symptom related request, and therefore this should not take more than a few minutes per day. Findings from this project can help improve and enhance pharmacy practice by;

- Empowering pharmacists/pharmacy staff to have infection related self-care conversations with patients/customers
- Increase appropriate use of NHS resources and potentially reduce pressure on GPs
- Using available resources and signposting to sources of advice is an important aspect of Healthy Living Pharmacy champions as part of Making Every Contact Count (MECC).

If the study produces favourable results, it could be supported as part of the national "Stay Well this Winter" campaign in pharmacies.

The TARGET treating your infection leaflet is already in use and implemented for General Practice. By using it within pharmacy, it helps to reinforce the messages to patients/customers.

A payment of £50 per pharmacy will be available on completion of the study and submission of the data.

Can I withdraw from the study at any time?

Pharmacies may withdraw in writing or by emailing espaur@gov.uk from the study at any point, ideally before the 2nd of January 2018 when briefing pack and resources will be sent out.

You do not need to provide a reason for withdrawal and there will be no consequences as a result of withdrawal. Please note that data already provided may be included as part of the aggregate data.

What you'll find with this briefing note

Consultation Data Input Sheet (See Figure 1) and the TARGET leaflet (See Figure 2) below.

Figure 1. Consultation Data Input Sheet

Public Health England

Protecting and improving the nation's health

Consultation Data Input Sheet

Name of Lead:

Pharmacy name:

Phone number/email address:

Please add data to PharmOutcomes as soon as possible

Date of consultation	Age group Enter age group code (e.g. C, T, A, E*)	Gender	Type of Respiratory Tract Infection (Please tick appropriately)					OTC recommended? (Y/N)	Advised to see GP/other service? (Please include information on reason for referral to GP*)	Referred to Pharmacist (Y/N/NA)	Self-care advice given?	Written information provided (Y/N. If Yes, name)
			Middle ear infection	Sore throat	Common cold	Sinusitis	Cough					
1.												
2.												
3.												
4.												
5.												
6.												
7.												
8.												
9.												
10.												

Page ___ of ___

Contact details: Dr Diane Ashiru-Oredope, Dr Chaamala Klinger via espaur@phe.gov.uk

Figure 2. TARGET leaflet

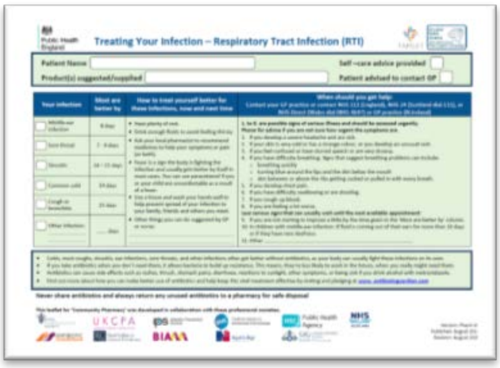

Who do I contact in the event of a complaint?

In the event of a complaint, please contact the AMR programme at espaur@gov.uk.

Thank you for taking the time to read this briefing note.

Antimicrobial stewardship and self-care advice in pharmacies (AMSAP) study

Briefing Note- Group B (CONTROL GROUP)

About the project

Tackling antimicrobial resistance (AMR) is a key public health priority. Inappropriate use and overuse of antimicrobials such as antibiotics is a major driver of antimicrobial resistance. Community Pharmacy teams have a key role in contributing to public health and promoting healthy living with the public. The project aims to assess the use of a resource as an integrated core part of community pharmacy practice for two weeks. This intervention will be evaluated by a randomised controlled trial. It is hoped that this project will generate evidence on the vital role played by Community Pharmacy Teams within public health. This project will also help provide evidence and contributes to Healthy Living Pharmacies, Making Every Contact Count and improving quality. Pharmacies can use the data collection as evidence of audit.

Who is doing the study?

The research leads for this study are Drs xxxxxx, and xxxxxxx. It is funded by the Public Health England Antimicrobial Resistance Programme and will take place in the South West of England and in Southwark.

What do you need to do?

- Collect some simple data on all conversations about self-care advice relating to patients/customers who come into the pharmacy with symptom related queries on
    - Middle ear infection
    - Sore throat
    - Common cold/runny nose
    - Sinusitis
    - Cough or bronchitis

  during the two weeks beginning 22nd and 29th January 2018. This should take a few minutes per day as there is no data being collected that you wouldn't have already

obtained as part of normal conversations with the patient/customer. A Consultation Data Input Sheet has been provided to aid data collection and data should be submitted using the PharmOutcomes Portal.

- Complete questionnaires about your experience of giving self-care advice to people with common infections which should take no more than 10 minutes.

Who has been invited to participate?

- All patient/customer facing staff in pharmacies in Avon, Cornwall, Devon, Gloucestershire, Somerset and Southwark are invited to take part.

Which patients/customers are included in the study?

Any patient/customer coming to your pharmacy who asks you for advice with any of the following infections:

- Middle ear infection
- Sore throat
- Common cold/runny nose
- Sinusitis
- Cough or bronchitis

Which patients/customers are not included in the study?

- Patients/customers coming to the pharmacy with the above respiratory tract infections who have a prescription for antibiotics from their GP
- Patients/customers who buy a product will be excluded if they do not seek advice on managing their illness
- Patients/customers coming to the pharmacy for any other reason such as self-care for other conditions (for example back pain, constipation, etc.)

What are the timelines for the project?

Action	Commence week beginning
You will receive briefing notes and Consultation Data Input Sheets	2nd Jan 2018
Complete Consultation Data Input Sheet for 2 weeks	22nd and 29th Jan 2018
Evaluation questionnaires will be sent you	5th Feb 2018
Deadline for questionnaire completion	18th Feb 2018

What should I be aware of when completing the Consultation Data Input Sheet?

A Consultation Data Input Sheet has been provided to aid data collection and data should be submitted using the PharmOutcomes Portal. The key definitions and explanations are listed below. These definitions can also be found in the Consultation Data Input Sheet.

Consultation: Any verbal exchange with a patient/customer about symptoms of the common infections listed above. This may include minimal contact such as a simple request for a cough medicine

Age group code: C: Child; T: Teenager; A: Adult; E: Elderly

OTC Meds: Over the counter medications given/purchased.

Advised to see GP/ Out of hours/ A and E-

"No" indicates either

- you did not mention the GP or
- you advised the patient/customer not to see the GP if they asked you or
- you suggested they should see them only if things do not improve.

"Yes" indicates you suggested the patient/customer should contact the GP straight away.

Yes GP= GP

Yes OOH service= OOH

Yes A and E= AE

Self-care advice given-“Yes” indicates you suggested simple measure that patients/customers can take to care for themselves; for example taking plenty of rest. Dosage or compliance advice is not included. “No” indicates no advice on these matters was offered.

Leaflet given: Did you give a leaflet about antibiotics, infections or self-care for them to take home? If “yes”, what is the name of the leaflet you used?

When should I complete the Consultation Data Input Sheet?

It is best to complete the data input each time you have a consultation. This will help to capture the data accurately and ensure that all of the information is captured. Data should be submitted using the PharmOutcomes Portal. Remember to ensure that no patient/customer-sensitive information, such as names or addresses are included. All data should be added to PharmOutcomes by 9th February 2018.

When should I complete the Post-Study Questionnaire?

Please complete the questionnaire as soon as possible when you receive it and before the 18th of February. **If you have been away on annual leave during the study period (22nd Jan to 4th Feb 2018), please do not complete the questionnaire.**

Will the information obtained in the study be confidential?

- This study has been reviewed by the PHE ethics and government group. In accordance with the Data Protection Act 1998, data will be kept confidential as follows: Data will be collected via questionnaires and data entry forms. Data will be stored on PharmOutcomes and PHE secure servers. No identifiable data will be collected.

What will happen to the results of the study?

- Summary of findings will be included in the 2018 national English Surveillance Programme for Antimicrobial Utilisation and Resistance (ESPAUR) report. Full findings will be submitted for peer review publication. We will provide a 1 page summary of findings to all participating pharmacies.

Do I have to take part?

- Taking part is entirely voluntary, and all participants will be asked to sign a consent form.

What are the advantages/benefits and disadvantages/risks of taking part?

The data being collected should be part of a normal consultation with the patient/customer who comes in with a symptom related request, and therefore this should not take more than a few minutes per day. Findings from this project can help improve and enhance pharmacy practice by;

- Empowering pharmacists/pharmacy staff to have infection related self-care conversations with patients/customers
- Increasing appropriate use of NHS resources and potentially reducing pressure on GPs
- Using available resources and signposting to sources of advice. This is an important aspect of Healthy Living Pharmacy champions as part of Making Every Contact Count (MECC).

If the study produces favourable results, it could be supported as part of the national “Stay Well this Winter” campaign in pharmacies.

A payment of £50 per pharmacy will be available on completion of the study and submission of data.

Can I withdraw from the study at any time?

Pharmacies may withdraw in writing or by emailing espaur@gov.uk from the study at any point, ideally before the 2nd of January 2018 when briefing pack and resources will be sent out.

You do not need to provide a reason for withdrawal and there will be no consequences as a result of withdrawal. Please note that data already provided may be included as part of the aggregate data.

What you'll find with this briefing note

Consultation Data Input Sheet (See Figure 1)

Figure 1. Consultation Data Input Sheet

Public Health England

Consultation Data Input Sheet

Name of Lead:

Pharmacy name:

Phone number/email address:

Please add data to PharmOutcomes as soon as possible

Date of consultation	Age group Enter age group code (e.g. C, T, A, E*)	Gender	Type of Respiratory Tract Infection (Please tick appropriately)					OTC recommended? (Y/N)	Advised to see GP/other service? (Please include information on reason for referral to GP*)	Referred to Pharmacist (Y/N/NA)	Self-care advice given?	Written information provided (Y/N. If Yes, name)
			Middle ear infection	Sore throat	Common cold	Sinusitis	Cough					
1.												
2.												
3.												
4.												
5.												
6.												
7.												
8.												
9.												
10.												

Page ___ of ___ Contact details: Dr Diane Ashiru-Oredope, Dr Chaamala Klinger via espaur@phe.gov.uk

Who do I contact in the event of a complaint?

In the event of a complaint, please contact the AMR programme at espaur@gov.uk.

Thank you for taking the time to read this briefing note.

Public Health England
Protecting and improving the nation's health

Supplementary Information 3: Consultation Data Input Sheet

Name of Lead:

Pharmacy name:

Phone number/email address:

Please add data to PharmOutcomes as soon as possible

Date of consultation	Age group Enter age group code (e.g. C, T, A, E*)	Gender	Type of Respiratory Tract Infection (Please tick appropriately)					OTC recommended? (Y/N)	Advised to see GP/other service? (Please include information on reason for referral to GP*)	Referred to Pharmacist (Y/N/NA)	Self-care advice given?	Written information provided (Y/N. If Yes, name)
			Middle ear infection	Sore throat	Common cold	Sinusitis	Cough					
1.												
2.												
3.												
4.												
5.												
6.												
7.												
8.												
9.												
10.												

Public Health England

Protecting and improving the nation's health

*Reasons for referral (free text)

Notes and key code

Consultation: Any verbal exchange with a patient about symptoms of the common infections listed above. This may include minimal contact such as a simple request for a cough medicine

****Age group code: C: Child; T: Teenager; A: Adult; E: Elderly***

OTC Meds- Over the counter medications given/purchased.

Advised to see GP/ Out of hours/ A and E-

"No" indicates either

- you did not mention the GP or
- you advised the patient not to see the GP if they asked you or
- you suggested they should see them only if things do not improve.

"Yes" indicates you suggested the patient should contact the GP straight away.

Yes GP= GP

Yes OOH service= OOH

Yes A and E= AE

Self-care advice given-"Yes" indicates you suggested simple measure that patients can take to care for themselves; for example taking plenty of rest. Dosage or compliance advice is not included. "No" indicates no advice on these matters was offered.

Leaflet given- Did you give a leaflet about antibiotics, infections or self-care for them to take home? If "yes", what is the name of the leaflet you used?

Supplementary Information 4: Behavioural assessment questionnaire

Date of completion:

This questionnaire relates only to patients/customers presenting with symptoms of common infections such as <u>middle ear infections, sore throats, common colds, sinusitis coughs and bronchitis.</u>

1. **Your role (please tick most appropriate box:**
   ☐Pharmacist
   ☐Pharmacist manager
   ☐Pharmacy owner
   ☐Locum pharmacist
   ☐Pharmacy technician
   ☐Dispenser
   ☐Counter assistant
   ☐Other pharmacy staff (please specify)

2. **Which type of pharmacy do you work in?**
   ☐Independent
   ☐Multiple - Boots
   ☐Multiple - Lloyds
   ☐Multiple - Weldricks
   ☐Multiple - Other
   ☐Health Centre

3. **Did you work in more than 1 pharmacy in the past two weeks?**
   Yes/No

4. **Have you used any patient/customer information leaflets in the last two weeks for patients/customers presenting with common infections?**
   ☐No
   ☐Yes, the TARGET leaflet
   ☐Yes Keep Antibiotics Working leaflet
   ☐ 'Managing your child's cough' leaflet
   ☐'When should I worry?' leaflet
   ☐Yes, other
   Free text………………………..

5. **Have you been involved in an Antibiotic stewardship project during the last year?**
   Yes/ No
   If yes, please describe below (free text)
   Name:
   Month/Year:

6. **Have you chosen a pledge on Antibiotic Guardian.com?**
   Yes/ No

7. **Are you aware of any local primary care antibiotic guidance / formulary? (please tick most appropriate response)**
   ☐Yes and this pharmacy uses it regularly
   ☐Yes but this pharmacy does not regularly refer to it
   ☐No

Self-care advice

For each of the following statements, please rate your agreement or disagreement with the following statements where 1=Strongly disagree and 5=Strongly agree

Capability

8. I know the signs and symptoms which should prompt a patient to get urgent advice from a health professional.
9. I know how long the symptoms of common infections usually last.
10. I know what self-care advice to give to patients with common infections.
11. I know what antibiotic resistance is.
12. There is a connection between giving self-care advice and reducing antibiotic resistance.

Motivation

13. I find it easy to give self-care advice to patients/customers who present with common infections.
14. I am confident that I can give self-care advice even when I feel under pressure.
15. I find it difficult to explain to patients/customers why they should not have antibiotics for common infections.
16. I believe that patients/customers will make fewer GP appointments for common infections as a result of my self-care advice.
17. I have a key role in helping to control antibiotic use.
18. Patients/customers will make GP appointments for common infections regardless of what I say.
19. It is important that I give self-care advice for common infections.

Opportunity

20. I have easy access to the materials I need to give self-care advice.
21. I don't get the opportunity to give all the advice I want to give because of other time pressures.
22. I feel supported to give self-care advice to patients/customers.
23. When patients/customers are in a hurry to leave the pharmacy they miss out on receiving self-care advice.

Behaviour

These questions refer to the two week trial period, 22nd January to 4th February 2018, and to patients/ customers presenting with common infections (such as Middle ear infection, Sore throat, Common cold/runny nose, Sinusitis and Cough or Bronchitis) during that time.

24. On a typical day, how often did you have a conversation about self-care with a patient/ customer?
☐Never
☐Rarely
☐Sometimes
☐Often
☐Very often

25. On a typical day, how often did you give out self-care resources, information or advice to a patient/ customer?
☐Never
☐Rarely
☐Sometimes
☐Often
☐Very often

26. On a typical day, how often would you have liked to give a patient/ customer self-care resources, information or advice but were not able to?
☐Never
☐Rarely
☐Sometimes
☐Often
☐Very often

27. On a typical day, how often did you refer a patient/ customer presenting with symptoms of a common infection to the GP?
☐Never
☐Rarely

☐Sometimes
☐Often
☐Very often

28. On a typical day, please estimate how many times you had a conversation about self-care with a patient/ customer.
☐Never
☐Rarely
☐Sometimes
☐Often
☐Very often

29. On a typical day, please estimate how many times you had a conversation about self-care with a patient/ customer.

30. On a typical day, please estimate how many times you gave out self-care advice to a patient/ customer.

31. On a typical day, please estimate how many times you would have liked to have given a patient/ customer self-care advice but were not able to.

32. On a typical day, please estimate how many times you referred a patient/ customer presenting with symptoms of a common infection to the GP.

WEBINAR EVALUATION

1. Did you watch the webinar?
☐Yes, all of it
☐Yes, most of it
☐Yes, some of it
☐None of it

For each of the following statements, please rate your agreement or disagreement with the following statements where 1= Strongly disagree and 5=Strongly agree

1. I found the webinar useful.
2. Watching the webinar increased my confidence in participating in the study.
3. The webinar gave me a greater appreciation for my personal role in antimicrobial stewardship
4. The webinar increased my understanding of patients' expectations and behaviour
5. The webinar increased my understanding of the importance of self-care for patients/customers who present with common infections.
6. The webinar increased my confidence in my ability to give good self-care advice.
7. The webinar made me more confident in explaining the connection between antibiotic use and antibiotic resistance to patients/customers.
8. Watching the webinar has made me more committed to giving self-care advice.
9. Since watching the webinar, I intend to give more self-care advice.
10. Please describe how you feel that the webinar could be improved? (Optional Question) (Free text field)

DEMOGRAPHICS

1. Your age:
☐Under 25
☐25-24

☐35-44
☐45-54
☐55+

2. Gender:
   ☐Male
   ☐Female
   ☐ Other
   ☐ Prefer not to say

3. Number of years of pharmacy experience:
4. Have you undertaken any training or continued professional development in antimicrobial resistance or a related topic?

☐No
☐Yes; please specify
Free text………………………..

Supplementary Information 5: Process evaluation questionnaire

To be sent to lead contact in each pharmacy

Process evaluation- completely voluntary
We would be grateful if you can answer a few additional questions to help us evaluate the process of this project.

Please provide contact details of the member of staff completing the process evaluation on behalf of the pharmacy:
____

1. What percentage of your staff participated in the data collection?

Evaluation of resources

2. On a scale of 1-5 (1 being poor and 5 being Excellent) please identify:

A. How well informed your pharmacy team was regarding this project?
B. How easy it was to identify resources for this project?

Project data recording

3. When did your staff usually complete the Consultation Data Input Table?
   ☐End of each shift
   ☐After every relevant consultation
   ☐At end of 2 week data collection period
   ☐Ad-hoc
   ☐None

4. What percentage of relevant consultations do you think were captured in the consultation data input table?
   ☐Up to 25%
   ☐More than 25% but less than 50%
   ☐75%
   ☐100%

5. If fewer than 100%, what were the reasons for your staff not being able to complete the table? (Tick all that apply).
   ☐Did not know how to complete it
   ☐Did not think it was worth completing
   ☐Did not have the information needed to complete it
   ☐Other things were more important
   ☐Too busy
   ☐Forgot to complete it
   ☐Did not have the table to complete
   ☐Could not recall the information to complete
   ☐Other, please state
   Free text………………………..

6. I would be interested in using a similar leaflet giving information about self-care and prevention of suspected urinary tract infections (scale 1-5 where 1= not interested 5= very interested)

7. Are there any extra support needs or training you would like from NHS England/Public Health England regarding managing infections?

Options: (please select any that apply)
☐Webinars
☐Online training

☐Face to face training
☐No further support required
☐Resources to support patient information around compliance
☐Easier access to local antibiotic guidance
☐Easier access to local use data
☐Easier access to local resistance guidance
☐Personalised visits to develop pharmacy plan
Other please state (free text box)…………………………………………………

8. Please tell us what worked well and what didn't work so well during the Antimicrobial stewardship and self-care advice in pharmacies (AMSAP) study (free text)

9. Would you or one of team be willing to take part in a short interview to further understand if there are any barriers or challenges?

Yes
No
If yes, please give contact details (free text)

Thank you for your support during this campaign and for making a positive impact to antibiotic microbial stewardship within your local community. We will be producing an evaluation of the project. If you would like to receive the final evaluation report, please provide us with an email address we can send this to.

Supplementary Figure 1: Summary of study activities

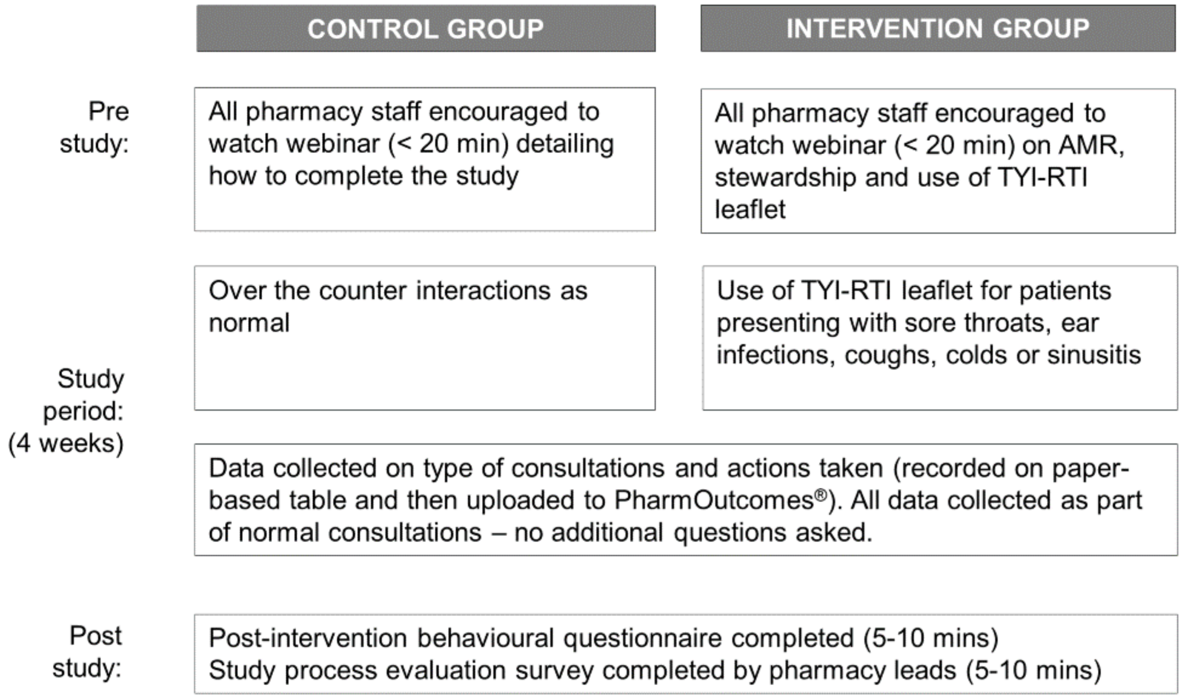

Supplementary Figure 2: Data collection and responses from pharmacies in intervention and control arms

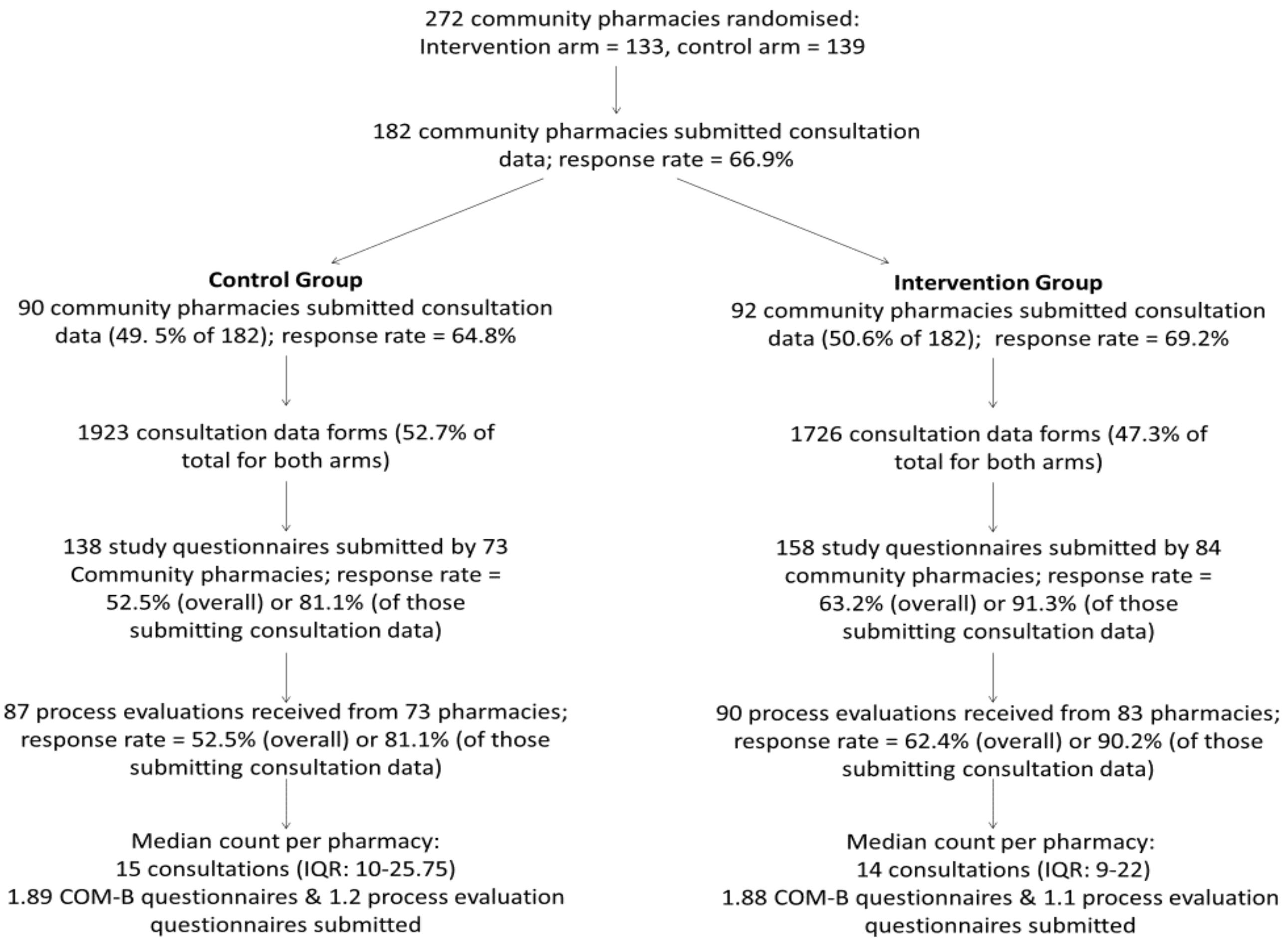

CONSORT 2010 checklist of information to include when reporting a randomised trial*

Section/Topic	Item No	Checklist item	Reported on page No
**Title and abstract**			
	1a	Identification as a randomised trial in the title	1
	1b	Structured summary of trial design, methods, results, and conclusions (for specific guidance see CONSORT for abstracts)	2
**Introduction**			
Background and objectives	2a	Scientific background and explanation of rationale	3-4
	2b	Specific objectives or hypotheses	4
**Methods**			
Trial design	3a	Description of trial design (such as parallel, factorial) including allocation ratio	4-8
	3b	Important changes to methods after trial commencement (such as eligibility criteria), with reasons	6-8
Participants	4a	Eligibility criteria for participants	5
	4b	Settings and locations where the data were collected	5
Interventions	5	The interventions for each group with sufficient details to allow replication, including how and when they were actually administered	5-8 and suppl information
Outcomes	6a	Completely defined pre-specified primary and secondary outcome measures, including how and when they were assessed	8
	6b	Any changes to trial outcomes after the trial commenced, with reasons	8
Sample size	7a	How sample size was determined	5
	7b	When applicable, explanation of any interim analyses and stopping guidelines	
Randomisation:			
Sequence generation	8a	Method used to generate the random allocation sequence	5-6
	8b	Type of randomisation; details of any restriction (such as blocking and block size)	6
Allocation concealment mechanism	9	Mechanism used to implement the random allocation sequence (such as sequentially numbered containers), describing any steps taken to conceal the sequence until interventions were assigned	6
Implementation	10	Who generated the random allocation sequence, who enrolled participants, and who assigned participants to interventions	6
Blinding	11a	If done, who was blinded after assignment to interventions (for example, participants, care providers, those	6

		assessing outcomes) and how	
	11b	If relevant, description of the similarity of interventions	
Statistical methods	12a	Statistical methods used to compare groups for primary and secondary outcomes	8
	12b	Methods for additional analyses, such as subgroup analyses and adjusted analyses	
**Results**			
Participant flow (a diagram is strongly recommended)	13a	For each group, the numbers of participants who were randomly assigned, received intended treatment, and were analysed for the primary outcome	8; Table 1
	13b	For each group, losses and exclusions after randomisation, together with reasons	
Recruitment	14a	Dates defining the periods of recruitment and follow-up	6-8
	14b	Why the trial ended or was stopped	
Baseline data	15	A table showing baseline demographic and clinical characteristics for each group	19 – table 1
Numbers analysed	16	For each group, number of participants (denominator) included in each analysis and whether the analysis was by original assigned groups	19
Outcomes and estimation	17a	For each primary and secondary outcome, results for each group, and the estimated effect size and its precision (such as 95% confidence interval)	19-24
	17b	For binary outcomes, presentation of both absolute and relative effect sizes is recommended	
Ancillary analyses	18	Results of any other analyses performed, including subgroup analyses and adjusted analyses, distinguishing pre-specified from exploratory	9; 21-24
Harms	19	All important harms or unintended effects in each group (for specific guidance see CONSORT for harms)	
**Discussion**			
Limitations	20	Trial limitations, addressing sources of potential bias, imprecision, and, if relevant, multiplicity of analyses	14
Generalisability	21	Generalisability (external validity, applicability) of the trial findings	14-15
Interpretation	22	Interpretation consistent with results, balancing benefits and harms, and considering other relevant evidence	12-15
**Other information**			
Registration	23	Registration number and name of trial registry	
Protocol	24	Where the full trial protocol can be accessed, if available	NA
Funding	25	Sources of funding and other support (such as supply of drugs), role of funders	16

*We strongly recommend reading this statement in conjunction with the CONSORT 2010 Explanation and Elaboration for important clarifications on all the items. If relevant, we also recommend reading CONSORT extensions for cluster randomised trials, non-inferiority and equivalence trials, non-pharmacological treatments, herbal interventions, and pragmatic trials. Additional extensions are forthcoming: for those and for up to date references relevant to this checklist, see www.consort-statement.org.
